# The prognostic significance of metabolic syndrome and weight loss in esophageal squamous cell carcinoma

**DOI:** 10.1038/s41598-018-28268-2

**Published:** 2018-07-04

**Authors:** Bowen Liu, Bo Cheng, Cong Wang, Pengxiang Chen, Yufeng Cheng

**Affiliations:** 1 Department of Radiation Oncology, Qilu Hospital of Shandong University, Jinan, Shandong P.R. China; 2 Department of Radiation Oncology, Shandong Provincial Cancer Hospital, Jinan, Shandong P.R. China

## Abstract

Our study aimed to investigate the association between metabolic syndrome and postoperative survival in patients with esophageal squamous cell carcinoma, and evaluate whether metabolic syndrome can predict the prognosis in esophageal cancer patients. The retrospective study reviewed 519 patients with esophageal squamous cell carcinoma who had received esophagetomy and lymphnode dissections in the Department of Thoracic Surgery, Qilu Hospital of Shandong University between January 2007 and December 2011. All patients were followed up until December 2016. The median follow-up time was 39.59 months (range 0.25–72 months). The 3-year and 5-year survival rate was 51.4% and 37.0%, respectively. Kaplan–Meier survival analysis revealed a significant correlation between OS and obesity (P = 0.000), weight loss (P = 0.000), diabetes (P = 0.001) and dyslipidemia (P = 0.030). Multivariate analysis indicated that advanced TNM staging (P = 0.007, HR: 1.760, 95% CI: 1.167–2.654) and more weight loss (P = 0.000, HR: 1.961, 95% CI: 1.697–2.267) were independent factors for adverse prognosis of esophageal squamous carcinoma patients. In contrast, diabetes was a protective factor in the prognosis of patients with esophageal cancer (P = 0.018, HR: 0.668, 95% CI: 0.478–0.933). Our findings suggest that TNM staging, weight changes and diabetes were independent predictors for the prognosis of esophageal cancer patients.

## Introduction

Esophageal cancer (EC) is one of the most common malignant gastrointestinal tumors in the world. With increasing incidence and mortality, esophageal cancer is the leading cause of death in China. The latest malignancy epidemiology data showed that the number of new cases was 477,900 people and the number of deaths was 375,000 people in China in 2015^1^. Esophageal cancer has two main subtypes: esophageal squamous cell carcinoma (ESCC) and esophageal adenocarcinoma (EAC)^2^. ESCC accounts for about 90% of cases of esophageal cancer worldwide^3^, which is predominant in Asia, Africa, and South America^4^. At present, the main treatment methods for esophageal squamous cell carcinoma include: surgical treatment, radiotherapy, chemotherapy, immunotherapy, comprehensive treatment and other treatment methods. In the early stage of esophageal cancer, there are no obvious clinical symptoms. Most of the patients are diagnosed in the middle or advanced stage. ESCC has high malignancy, rapid development process, poor therapeutic effect, and high rate of recurrence and metastasis. Although early detections and surgical techniques have improved, the five-year survival rate of esophageal cancer remains generally poor, according to the regional metastasis or distant metastasis^5^. Therefore, it is necessary to determine the factors that affect the postoperative prognosis and the recurrence or metastasis.

The metabolic syndrome (MS), including hyperglycaemia, dyslipidemia, obesityand hypertension, has become one of the major worldwide public-health challenges^6^. MS had been highlighted as a risk factor for ischemic heart disease and arteriosclerotic disease, it was also recently shown to be associated with several tumors^7–9^. Some studies have indicated that certain factors in MS were associated with risk of cancer, including liver, colorectal, bladder, pancreatic, breast and esophageal cancer^10–12^. Obesity was associated with an increased risk of EAC^12^ and a decreased risk of ESCC^13^. A prospective study included 1082 esophageal cancer showed that there was a strong inverse association between BMI and death from esophageal cancer^13^. Several studies have shown that preoperative weight loss in esophageal cancer resection might have a certain relationship with postoperative survival^14,15^.The evidence for the positive correlation between lipid metabolism and risk of esophageal cancer was presented^16^. There is now accumulating evidence that the MS may be not only risk factor for tumors but also cancer mortality marker^10^. However, the relationship between MS factor and prognosis of esophageal cancer is controversial. Some studies suggested that MS was associated with better prognosis of esophageal cancer^17^, while others failed to establish a connection^18^. Accordingly, the effects of MS and its related factors on overall survival (OS) and progression-free survival (PFS) of esophageal cancer need to be further discussed.

Therefore, we conducted this retrospective study to investigate the relationship between MS, weight loss and postoperative survival in patients with ESCC, and evaluate whether MS or weight loss can predict the prognosis in esophageal cancer patients.

## Results

### Patient characteristics

According to the classification of pathological types, there were 549 patients, including 519 patients with esophageal squamous cell carcinoma, 15 patients with esophageal adenocarcinoma, 10 patients with basal cell type squamous cell carcinoma and 5 patients with neuroendocrine carcinoma. Taking into account the number of samples, this experiment included only 519 patients with esophageal squamous cell carcinoma.

The clinicopathological characteristics of the patients are presented in Table 1. The median age at diagnosis was 62.08 years (ranged from 32 to 86 years), including 425 males (81.9%) and 94 females (18.1%). The median follow-up time was 39.59 months (range 0.25–72 months). The 3-year and 5-year survival rate was 51.4% and 37.0%, respectively. According to TNM stage, the number of patients in phase I, II and III was 73 (14.1%), 256 (49.3%), 190 (36.6%), separately. All patients underwent surgical treatment without receiving neoadjuvant chemotherapy or radiotherapy. About 59.3% of the patients received surgery alone, while the others received adjuvant postoperative therapy including radiotherapy (12.9%), chemotherapy (12.5%) and chemoradiotherapy (15.2%).

**Table 1:** Clinical and pathological characteristics of patients.

Characteristics	Classification	Patients (n)	Patients (percent)
Age	<60 years	201	38.7%
	≥60 years	318	61.3%
Gender	Male	425	81.9%
	Female	94	18.1%
Smoking	Ever	253	48.7%
	Never	266	51.3%
Drinking	Ever	218	42.0%
	Never	301	58.0%
Family history	Yes	33	6.4%
	No	486	93.6%
Location	Cervical	39	7.5%
	Upper	34	6.6%
	Middle	269	51.8%
	Lower	177	34.1%
Length	<4 cm	270	52.0%
	≥4 cm	249	48.0%
Differentiation	GX	16	3.1%
	G1	100	19.3%
	G2	204	39.3%
	G3	199	38.3%
T stage	Tis	16	3.1%
	T1	57	11.0%
	T2	152	29.3%
	T3	285	54.9%
	T4	9	1.7%
LNM	Yes	225	43.4%
	No	294	56.6%
N stage	N0	294	56.6%
	N1	130	25.0%
	N2	67	12.9%
	N3	28	5.4%
TNM	I	73	14.1%
	II	256	49.3%
	III	190	36.6%
Treatment	S	308	59.3%
	S + RT	67	12.9%
	S + CT	65	12.5%
	S + RCT	79	15.2%
BMI	<18.5	86	16.6%
	18.5~25	347	66.9%
	>25	86	16.6%
Weight loss	No	96	18.5%
	A little	89	17.1%
	Middle	239	46.1%
	Much	95	18.3%
Hypertension	No	410	79.0%
	Yes	109	21.0%
Diabetes	No	430	82.9%
	Yes	89	17.1%
Dyslipidemia	No	434	83.6%
	Yes	85	16.4%
HDL	<0.9	49	9.4%
	≥0.9	470	90.6%
TG	<1.7	475	91.5%
	≥1.7	44	8.5%
MS	No	466	89.8%
	Yes	53	10.2%

Abbreviations: LNM, lymph node metastasis; S, surgery; RT, radiotherapy; CT, chemotherapy; CRT, chemoradiotherapy; BMI, Body Mass Index; HDL, high density lipoprotein; TG, triglyceride; MS, metabolic syndrome.

There were 53 patients with metabolic syndrome and 466 patients with normal metabolic levels. The distribution of BMI was as follows: lower in 86 (16.6%) patients, normal in 347 (66.9%) patients, higher in 86 (16.6%) patients. 35.6% of patients had no significant changes in body weight during treatment, 46.1% of patients had middle weight loss, while 18.3% of patients lost weight significantly. In addition, the proportion of patients with hypertension, diabetes and dyslipidemia was 21.0%, 17.1% and 16.4%.

### Associations of BMI, weight loss, hypertension, diabetes, dyslipidemia and metabolic syndrome with clinicopathological characteristics

The relationship between clinicopathological features and metabolic syndrome was statistically analyzed and the results were shown in Table 2. The decrease of BMI level had a certain correlation with the progress of tumor differentiation grade (P = 0.010), N stage (P = 0.039), lymph node metastasis (P = 0.010) and TNM stage (P = 0.010). In addition, weight loss was positive associated with increased T stage (P = 0.047), N stage (P = 0.000), lymph node metastasis (P = 0.000) and TNM stage (P = 0.000). In addition, hypertension, diabetes, dyslipidemia and MS had no significant relationship with clinicopathological characteristics. A correlation analysis between patients with esophageal cancer who are accompanied with obesity, diabetes, dyslipidemia, and hypertension and weight loss can be found as Supplementary Table S1.

**Table 2:** Clinicopathological characteristics of 519 esophageal squamous cell carcinoma patients grouped by BMI, weight loss, hypertension, diabetes, dyslipidemia and metabolic syndrome.

Characteristics	BMI			Weight loss				Hypertension		Diabetes		Dyslipidemia		MS	
	<18.5	18.5~25	>25	No	A little	Middle	Much	No	Yes	No	Yes	No	Yes	No	Yes
	86	347	86	96	89	239	95	410	109	430	89	434	85	466	53
**Differentiation**															
GX	4	6	6	5	2	8	1	13	3	13	3	12	4	14	2
G1	12	63	25	19	25	43	13	75	25	81	19	85	15	86	14
G2	40	136	28	39	35	90	40	157	47	162	42	168	36	182	22
G3	30	142	27	33	27	98	41	165	34	174	25	169	30	184	15
P value	**0**.**010**			0.238				0.339		0.178		0.684		0.350	
**T stage**															
Tis	1	8	7	6	4	5	1	13	3	12	4	12	4	12	4
T1	5	40	12	16	15	19	7	45	12	50	7	45	12	51	6
T2	22	106	24	30	27	67	28	122	30	119	33	131	21	135	17
T3	56	187	42	44	42	142	57	223	62	243	42	238	47	260	25
T4	2	6	1	0	1	6	2	7	2	6	3	8	1	8	1
P value	0.060			**0**.**047**				0.990		0.153		0.611		0.327	
**LNM**															
Yes	49	146	30	25	29	122	49	178	47	192	33	190	35	204	21
No	37	201	56	71	60	117	46	232	62	238	56	244	50	262	32
P value	**0**.**010**			**0**.**000**				0.956		0.189		0.658		0.563	
**N stage**															
N0	37	201	56	71	60	117	46	232	62	238	56	244	50	262	32
N1	24	87	19	15	19	74	22	98	32	108	22	106	24	115	15
N2	17	44	6	8	5	38	16	58	9	60	7	58	9	64	3
N3	8	15	5	2	5	10	11	22	6	24	4	26	2	25	3
P value	**0**.**039**			**0**.**000**				0.342		0.387		0.446		0.423	
**TNM**															
I	6	48	19	21	20	24	8	55	18	65	8	58	15	64	9
II	39	172	45	57	47	108	44	201	55	202	54	215	41	227	29
III	41	127	22	18	22	107	43	154	36	163	27	161	29	175	15
P value	**0**.**010**			**0**.**000**				0.575		0.052		0.570		0.403	

### The relationship between BMI, weight loss, diabetes, dyslipidemia and OS, PFS of ESCC

**Figure 1:**
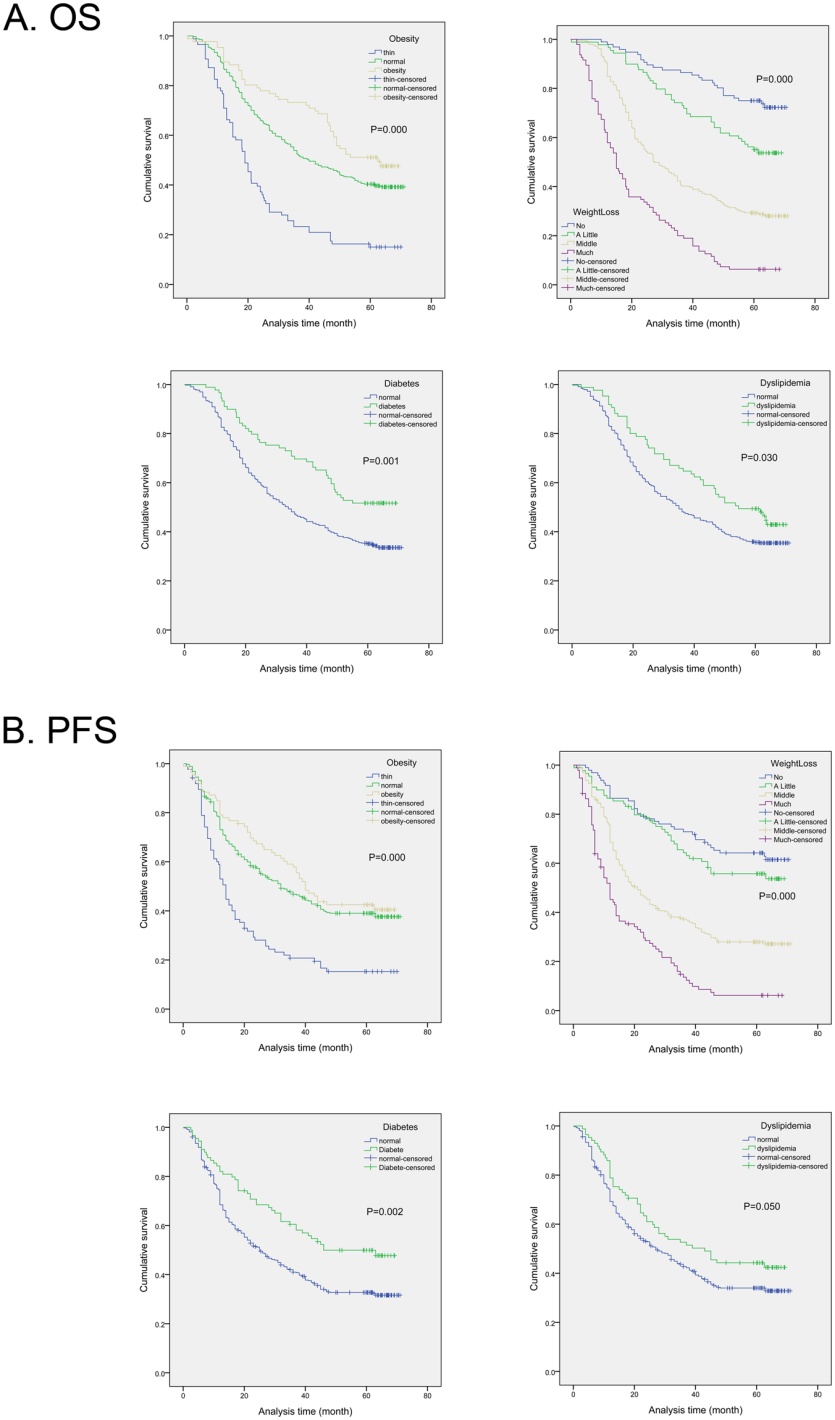
Kaplan-Meier analysis for overall survival (OS) and progression-free survival (PFS) of esophageal squamous cell carcinoma patients according to metabolic syndrome components, including obesity, weight loss, diabetes and dyslipidemia.

In our research, Kaplan–Meier survival analysis revealed a correlation between BMI, weight loss, diabetes, dyslipidemia and OS, PFS of esophageal cancer. As shown in Fig. 1, Kaplan-Meier curves showed that ESCC patients with obesity had significantly longer OS (P < 0.001) and PFS (P < 0.001). Furthermore, patients with middle or more weight loss, regardless of OS or PFS, were significantly shorter than patients with little body weight changes during treatment (OS, P < 0.001; PFS, P < 0.001). Compared with people in normal blood glucose level, esophageal cancer patients with diabetes showed a better prognosis in OS (P = 0.001), and PFS (P = 0.002). In the same way, dyslipidemia patients had longer survival in OS (P = 0.030) than those in normal blood lipids, although there was no statistical difference in PFS (P = 0.050). However, hypertension had no relationship with the prognosis of patients with esophageal squamous cell carcinoma.

### 1-year and 3-year survival of patients with BMI, weight loss, diabetes, dyslipidemia

**Figure 2:**
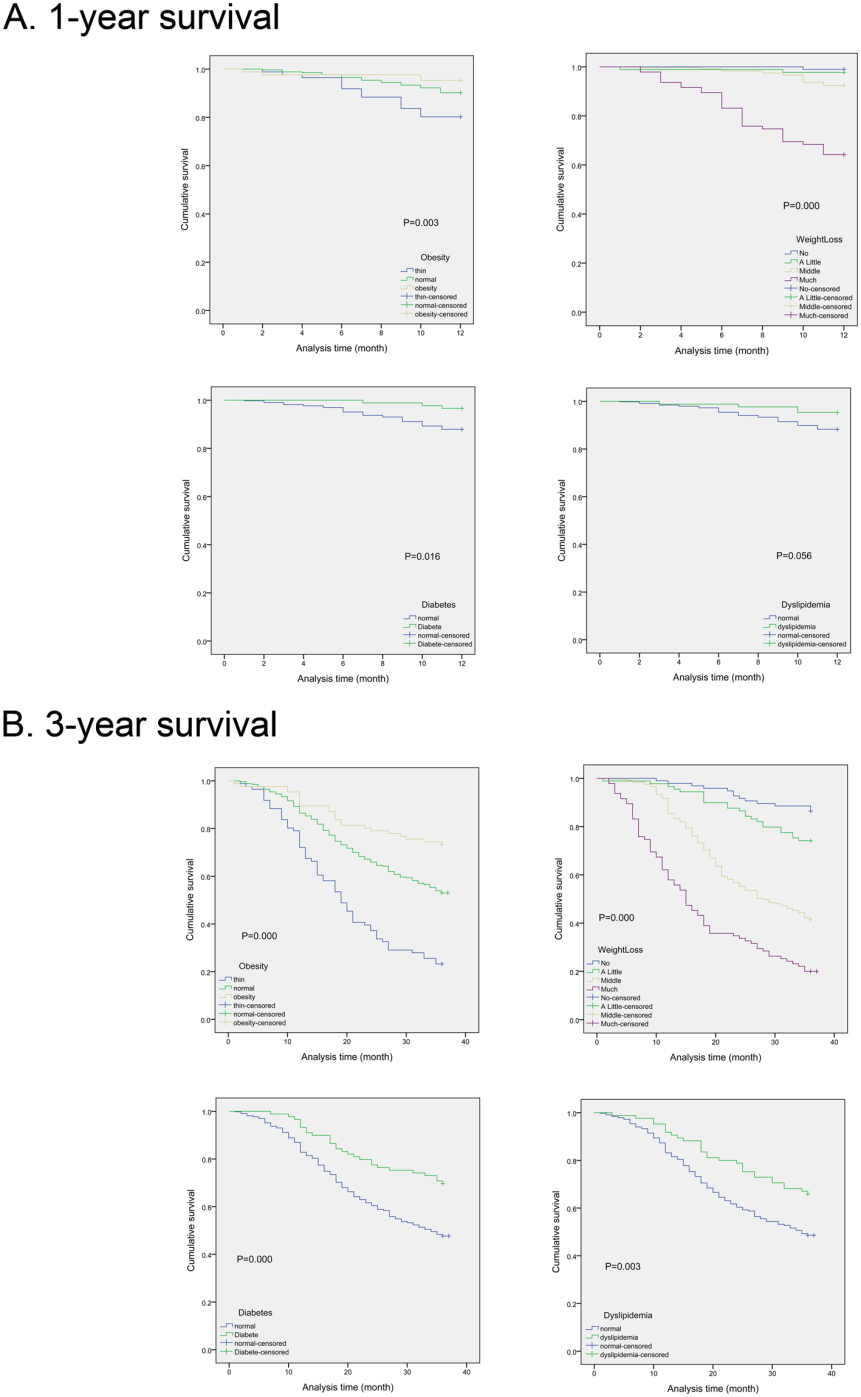
The association between obesity, weight loss, diabetes, dyslipidemia and 1-year, 3-years survival in esophageal cancer patients.

In the research, we detected the effects of BMI, weight loss, diabetes, dyslipidemia on 1-year and 3-year survival rate. It was showed in Fig. 2 that patients with obesity (1-year, P = 0.003; 3-year, P = 0.000), weight loss (1-year, P = 0.000; 3-year, P = 0.000), diabetes (1-year, P = 0.016; 3-year, P = 0.000) and dyslipidemia (1-year, P = 0.056; 3-year, P = 0.003) had better prognosis compared with normal metabolism patients, whether in 1-year OS or in 3-year OS.

### Univariate and multivariate analyses

The univariate analysis of suvival was exhibited in Table 3. There was a significant correlation between OS and gender (P = 0.019), tumor length (P = 0.008), tumor differentiation (P = 0.002), T stage (P = 0.000), LNM (P = 0.000), N stage (P = 0.000), TNM stage (N = 0.000), obesity (P = 0.000), weight loss (P = 0.000), diabetes (P = 0.001) and dyslipidemia (P = 0.032). The tumor location, treatment, hypertension, HDL, TG, MS and other factors did not have statistical significance with OS. In addition, univaviate analysis revealed a relationship between tumor length (P = 0.003), tumor differentiation (P = 0.002), T stage (P = 0.000), LNM (P = 0.000), N stage (P = 0.000), TNM stage (N = 0.000), treatment (P = 0.009), obesity (P = 0.000), weight loss (P = 0.000), diabetes (P = 0.002) and PFS time.

**Table 3:** Univariate analysis of prognostic factors in esophageal squamous cell carcinoma with respect to overall survival and progression-free survival.

Characteristics	Classification	OS			PFS		
		HR	95% CI	P value	HR	95% CI	P value
Age	<60 years	1.000	Ref.				
	≥60 years	1.141	0.911–1.430	0.250	1.194	0.953–1.495	0.123
Gender	Male	1.000	Ref.				
	Female	0.695	0.513–0.941	**0**.**019**	0.769	0.574–1.029	0.078
Smoking	Ever	1.000	Ref.				
	Never	0.869	0.699–1.079	0.203	0.939	0.757–1.165	0.566
Drinking	Ever	1.000	Ref.				
	Never	0.908	0.730–1.130	0.386	1.055	0.847–1.314	0.632
Family history	No	1.000	Ref.				
	Yes	1.081	0.701–1.667	0.723	1.056	0.685–1.627	0.805
Location	Cervical	1.000	Ref.				
	Upper	0.699	0.395–1.236	0.218	0.735	0.410–1.317	0.301
	Middle	0.756	0.503–1.137	0.179	0.892	0.591–1.347	0.587
	Lower	0.812	0.534–1.234	0.328	0.831	0.542–1.275	0.397
Length	<4 cm	1.000	Ref.				
	≥4 cm	1.341	1.079–1.666	**0**.**008**	1.388	1.118–1.722	**0**.**003**
Differentiation	GX	1.000	Ref.				
	G1	3.309	1.033–10.605	**0**.**044**	2.422	0.874–6.711	0.089
	G2	4.368	1.390–13.728	**0**.**012**	3.361	1.242–9.094	**0**.**017**
	G3	6.268	1.997–19.674	**0**.**002**	4.788	1.772–12.934	**0**.**002**
T stage	Tis	1.000	Ref.				
	T1	1.602	0.467–5.498	0.454	2.326	0.696–7.773	0.170
	T2	4.298	1.360–13.581	**0**.**013**	4.935	1.564–15.573	**0**.**006**
	T3	6.530	2.088–20.423	**0**.**001**	6.283	2.008–63.112	**0**.**002**
	T4	14.681	3.888–55.439	**0**.**000**	17.038	4.600–63.112	**0**.**000**
LNM	No	1.000	Ref.				
	Yes	2.901	2.322–3.626	**0**.**000**	2.584	2.074–3.220	**0**.**000**
N stage	N0	1.000	Ref.				
	N1	2.548	1.973–3.291	**0**.**000**	2.220	1.717–2.869	**0**.**000**
	N2	3.188	2.338–4.348	**0**.**000**	2.880	2.115–3.922	**0**.**000**
	N3	4.621	3.045–7.012	**0**.**000**	4.311	2.863–6.490	**0**.**000**
TNM	I	1.000	Ref.				
	II	2.992	1.807–4.955	**0**.**000**	2.437	1.556–3.816	**0**.**000**
	III	8.180	4.952–13.509	**0**.**000**	5.681	3.626–8.900	**0**.**000**
Treatment	S	1.000	Ref.				
	S + RT	1.063	0.688–1.643	0.783	1.248	0.788–1.976	0.345
	S + CT	0.858	0.396–1.856	0.697	1.144	0.525–2.493	0.735
	S + RCT	1.288	0.797–2.082	0.302	1.607	1.010–2.604	**0**.**009**
BMI	<18.5	1.000	Ref.				
	18.5~25	0.455	0.348–0.595	**0**.**000**	0.489	0.373–0.641	**0**.**000**
	>25	0.328	0.225–0.478	**0**.**000**	0.421	0.292–0.606	**0**.**000**
Weight loss	No	1.000	Ref.				
	A little	1.905	1.165–3.114	**0**.**010**	1.271	0.808–2.000	0.299
	Middle	4.234	2.798–6.406	**0**.**000**	2.857	1.991–4.099	**0**.**000**
	Much	9.749	6.263–15.174	**0**.**000**	5.693	3.833–8.455	**0**.**000**
Hypertension	No	1.000	Ref.				
	Yes	0.845	0.643–1.111	0.227	0.828	0.630–1.087	0.174
Diabetes	No	1.000	Ref.				
	Yes	0.579	0.420–0.799	**0**.**001**	0.611	0.446–0.837	**0**.**002**
Dyslipidemia	No	1.000	Ref.				
	Yes	0.713	0.523–0.971	**0**.**032**	0.740	0.545–1.005	0.054
HDL	<0.9	1.000	Ref.				
	≥0.9	1.384	0.934–2.053	0.106	1.081	0.747–1.564	0.681
TG	<1.7	1.000	Ref.				
	≥1.7	0.756	0.499–1.145	0.187	0.700	0.458–1.069	0.099
MS	No	1.000	Ref.				
	Yes	0.687	0.463–1.018	0.062	0.738	0.501–1.088	0.125

The multivariate analysis was shown in Table 4. The results indicated that advanced TNM staging (P = 0.007, HR: 1.760, 95% CI: 1.167–2.654) and more weight loss (P = 0.000, HR: 1.961, 95% CI: 1.697–2.267) were independent factors for adverse prognosis of esophageal squamous carcinoma patients. In contrast, diabetes was a protective factor in the prognosis of patients with esophageal cancer (P = 0.018, HR: 0.668, 95% CI: 0.478–0.933). It was also shown that TNM stage (P = 0.008, HR: 1.643, 95% CI: 1.140–2.369), weight loss (P = 0.000, HR: 1.674, 95% CI: 1.462–1.917) and dibetes (P = 0.027, HR: 0.691, 95% CI: 0.499–0.958) were independently associated with PFS.

**Table 4:** Multivariate analysis of prognostic factors in esophageal squamous cell carcinoma with respect to overall survival and progression-free survival.

Characteristics	OS			PFS		
	HR	95% CI	P value	HR	95% CI	P value
Gender	0.760	0.554–1.040	0.087			
Length	0.955	0.764–1.194	0.685	0.875	0.701–1.093	0.240
Differentiation	1.137	0.973–1.328	0.105	1.141	0.980–1.329	0.090
T stage	1.211	0.954–1.536	0.116	1.119	0.901–1.390	0.311
LNM	1.095	0.676–1.773	0.713	0.998	0.638–1.563	0.994
N stage	1.093	0.884–1.352	0.410	1.114	0.901–1.377	0.318
TNM	1.760	1.167–2.654	**0**.**007**	1.643	1.140–2.369	**0**.**008**
Treatment				1.097	0.997–1.206	0.058
Obesity	0.823	0.667–1.014	0.067	0.891	0.724–1.097	0.277
Weight loss	1.961	1.697–2.267	**0**.**000**	1.674	1.462–1.917	**0**.**000**
Diabetes	0.668	0.478–0.933	**0**.**018**	0.691	0.499–0.958	**0**.**027**
Dyslipidemia	0.969	0.706–1.331	0.847			

### Subgroup analysis

**Figure 3:**
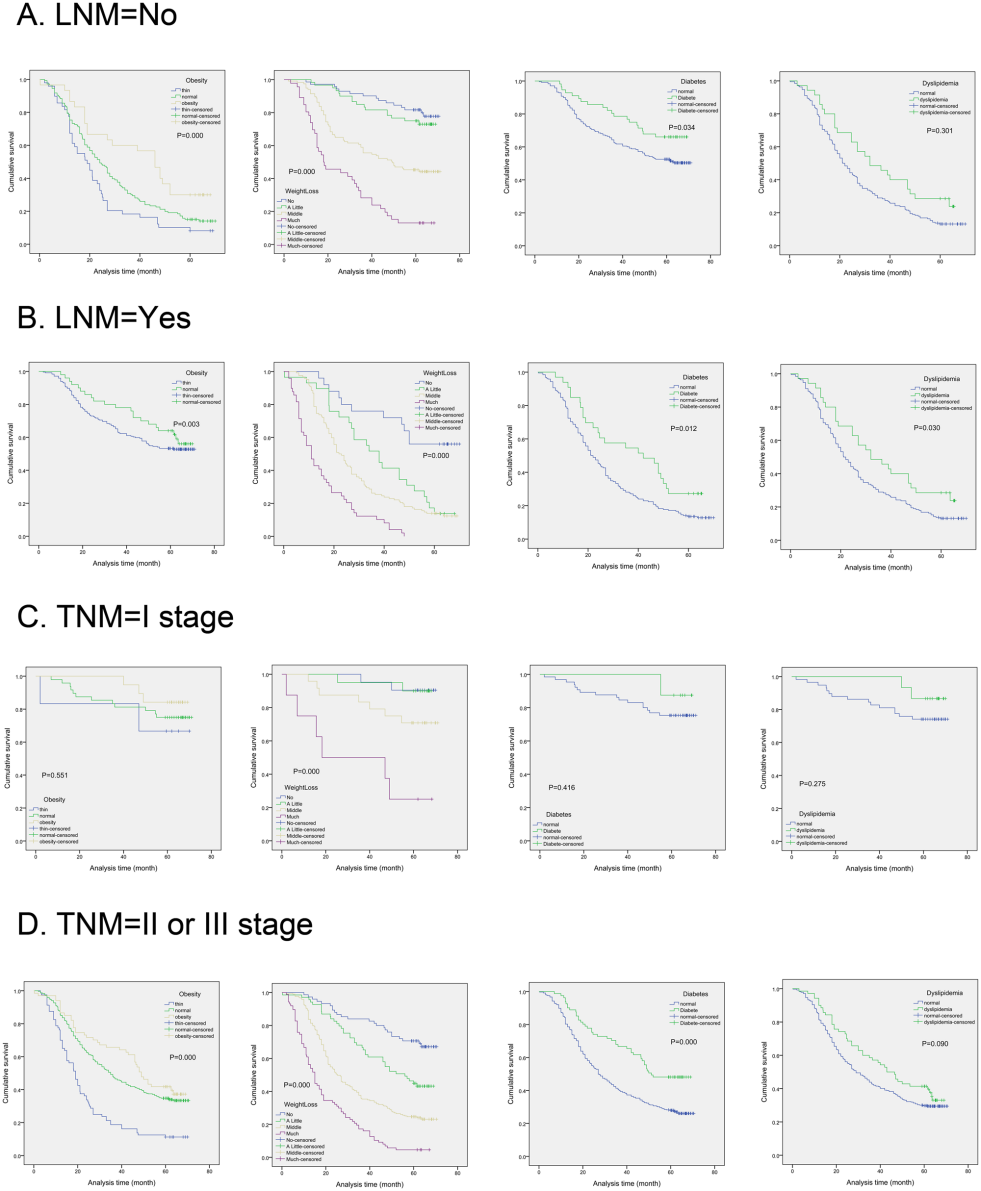
The effects of MS on survival in different subgroups. The effects of obesity, weight loss, diabetes and dyslipidemia on survival in different level of lymph node metastases (N0 vs N1–3) (**A**,**B**).The effects of obesity, weight loss, diabetes and dyslipidemia on survival in different TNM stages (TNM I stage vs TNM II-III stage) (**C**,**D**).

To detect the effects of BMI, weight loss, diabetes and dyslipidemia on survival in different level of lymph node metastases, we divided the patients into two subgroups (N0 vs N1–3). In N0 subgroup, obesity (P = 0.000), little weight loss (P = 0.000) and diabetes (P = 0.034) showed association with longer survival time, while dyslipidemia (P = 0.301) had no statistical significance (Fig. 3A and B). At the same time, it was indicated that BMI (P = 0.003), weight loss (P = 0.000), diabetes (P = 0.012) and dyslipidemia (P = 0.030) were all associated with survival in N1–3 subgroup.

As shown in Fig. 3C and D, we divided the patients into two subgroups (TNM I stage vs TNM II-III stage). It was revealed that BMI (P = 0.000), weight loss (P = 0.000) and diabetes (P = 0.000) showed association with longer OS in TNM II-III subgroup, while weight loss (P = 0.000) was related to survival time in subgroup TNM I stage.

**Figure 4:**
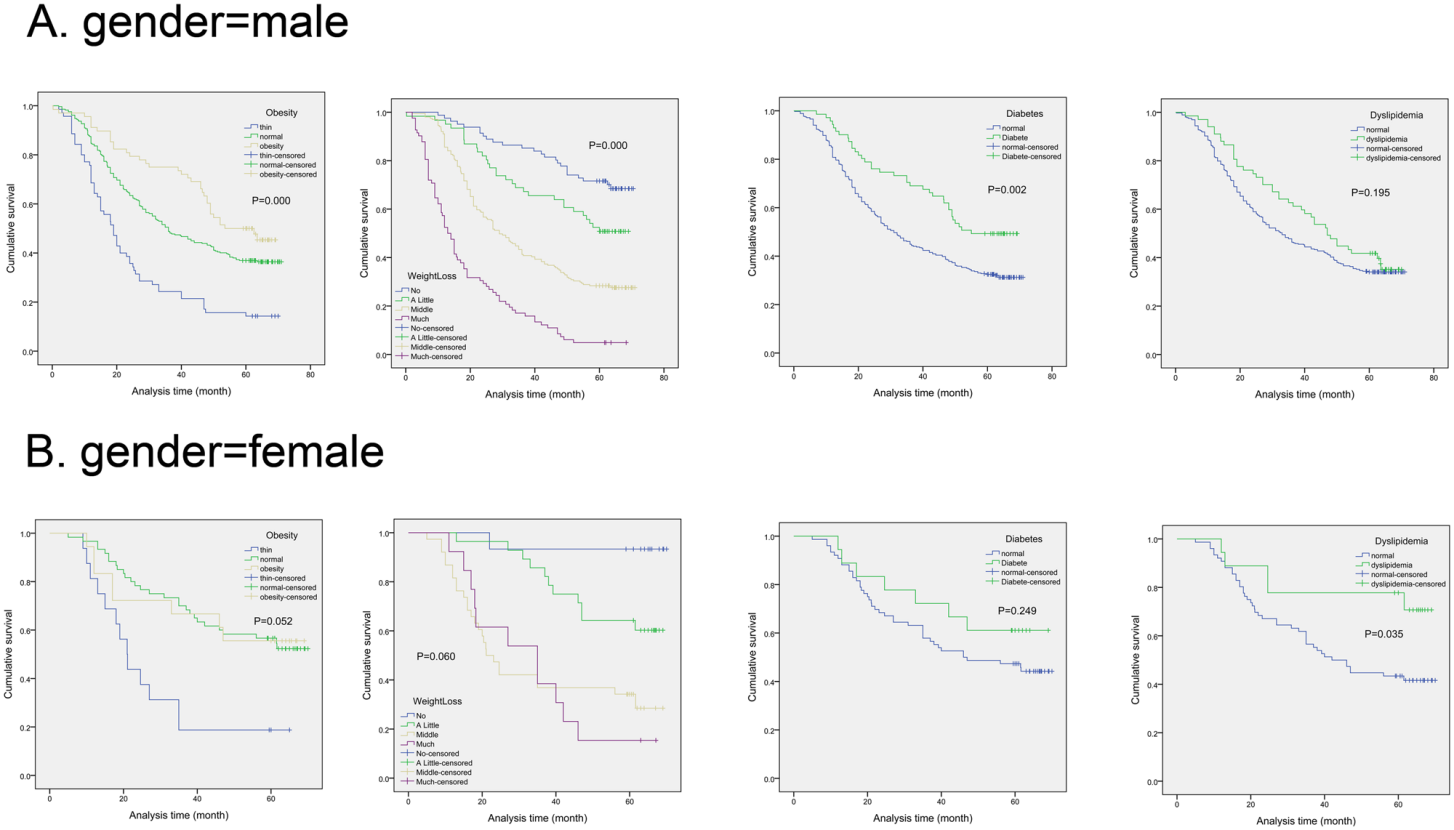
Kaplan–Meier analysis for the effects of obesity, weight loss, diabetes and dyslipidemia on survival in different gender categories (male vs female).

In male subgroup, obesity (P = 0.000), little weight loss (P = 0.000) and diabetes (P = 0.002) showed better survival compared with normal group in Fig. 4. Dislipidemia in female group showed better OS than normal patients (P = 0.035). Conversely, there were no significance in OS between patients accompanied by obesity (P = 0.052), little weight loss (P = 0.060), diabetes (P = 0.249) and ESCC patients with normal metabolic state in female subgroup. MS group showed better survival compared with normal group (P = 0.041) in males, while there was no significance between MS patients and normal ESCC patients in female subgroup (P = 0.846).

## Discussion

In this study, we investigated the prognostic value of MS in patients with resectable esophageal squamous cell carcinoma, and found significant correlation between OS and gender, tumor length, tumor differentiation, T stage, LNM, N stage, TNM stage, obesity, weight loss, diabetes and dyslipidemia. In addition, analysis revealed a relationship between tumor length, tumor differentiation, T stage, LNM, N stage, TNM stage, treatment, obesity, weight loss, diabetes and PFS time. The results indicated that advanced TNM staging and more weight loss were independent indicators for adverse prognosis of esophageal squamous carcinoma patients. In contrast, diabetes was a protective factor in the prognosis of patients with ESCC. It was also shown that TNM stage, weight loss and diabetes were independently associated with PFS time.

The metabolic syndrome, comprising obesity, dyslipidemia, hypertension, and hyperglycaemia, is a cluster of risk factors for cardiovascular disease and type 2 diabetes^6,19^. There is some evidence that MS may be associated with the risk of some common cancers^7–9^, including liver, colorectal, bladder, pancreatic, breast and esophageal cancer^10–12^. Some prospective cohort studies investigated the association between MS and the risk of the two dominating types of esophageal cancer, EAC and ESCC. Study found that obesity was associated with an increased risk of EAC^12^ and a decreased risk of ESCC^13^. In accordance with above study, Corley *et al*. detected that increasing abdominal diameter was strongly associated with an increased risk of esophageal adenocarcinoma but not esophageal squamous cell carcinoma^20^. The Me-Can project in Sweden conducted a prospective study and revealed an association between high blood pressure and risk of ESCC without adjusting alcohol consumption^21^. They also observed that MS was associated with EAC but not ESCC^21^. The evidence for the positive correlation between lipid metabolism and risk of esophageal cancer was presented^16^. Several studies indicated an association between high blood glucose and an increased risk of cancer overall^22,23^.

Although the relationship between MS and tumor risk was well studied, there is little research on the asscioation of metabolic factors and tumor mortality. It was indicated that MS might be an important prognostic factor for colorectal cancer. Patrizia *et al*. showed that metabolic syndrome at baseline emerged as an important prognostic factor for breast cancer recurrences^24^. However, there were different conclusions regarding the prediction value of MS in tumor prognosis. An analysis revealed no association between MS and prostate cancer risk. Moreover, it was proposed that the metabolic syndrome be considered as a high-risk state for certain types of cancer, such as colon cancer, and this relationship should be systematically explored across cancer types^25,26^.

Studies linking the metabolic syndrome to esophageal cancer are scarce. However, several studies had investigated the association between metabolic biomarkers and overall survival of esophageal cancer patients. A retrospective review was performed to show that MS was a significant and independent predictor for better survival in patients with resectable ESCC^27^. There was no apparent influence of any single component of MetS on OS^27^. Another study reported that low BMI and low fasting blood glucose were related to poor survival and BMI came to be stronger prognostic factors on lymph node-negative patients^28^. Our results were consistent with the above two researches. In our study, survival analysis revealed a correlation between BMI (P < 0.001), weight loss (P < 0.001), diabetes (P = 0.001), dyslipidemia (P = 0.030) and OS of esophageal cancer. In addition, our research also found that ESCC patients with obesity or little weight loss or diabetes had significantly longer PFS (obesity, P˂0.001; weight loss, P < 0.001; diabetes, P = 0.002). The multivariate analysis indicated that advanced TNM staging (P = 0.007, HR: 1.760, 95% CI: 1.167–2.654) and more weight loss (P = 0.000, HR: 1.961, 95% CI: 1.697–2.267) were independent factors for adverse prognosis of patients with ESCC. In contrast, diabetes was a protective factor in the prognosis of patients with esophageal cancer (P = 0.018, HR: 0.668, 95% CI: 0.478–0.933). It was also shown that TNM stage (P = 0.008, HR: 1.643, 95% CI: 1.140–2.369), weight loss (P = 0.000, HR: 1.674, 95% CI: 1.462–1.917) and dibetes (P = 0.027, HR: 0.691, 95% CI: 0.499–0.958) were independently associated with PFS. Nevertheless, there were conflicting results in the studies that had investigated whether MS influenced survival among esophageal cancer patients^13,17,18,29,30^. It was reported that BMI one year prior to diagnosis was not associated with esophageal adenocarcinoma survival in a study conducted in Australia^31^. Besides, a nationwide study in Sweden found that obese patients had a favourable prognosis in EAC, while lean patients had a better prognosis in ESCC, compared with normal weight patients^32^. The underlying mechanisms of the inverse relationship between MS and ESCC remained unclear.

In order to analyze the relationship between MS-related composition and survival at different levels, we carried out the subgroup analysis based on gender, lymph node metastasis and TNM stage. A study showed that HR of metabolic syndrome for ESCC mortality was statistically significant in male (HR: 1.45, 95% CI: 1.14–1.83, P:0.002), but not in female (HR: 1.46, 95% CI: 0.92–2.31, P: 0.107)^33^. Likewise, our research found that MS group showed better survival compared with normal group (P = 0.041) in males, while there was no significance between MS patients and normal ESCC patients in female subgroup (P = 0.846). In addition, we conducted subgroup analysis in accordance with lymph node metastasis and TNM staging. It was indicated that obesity (P = 0.000), little weight loss (P = 0.000) and diabetes (P = 0.034) showed association with survival time in N0 subgroup, while BMI (P = 0.003), weight loss (P = 0.000), diabetes (P = 0.012) and dyslipidemia (P = 0.030) were all associated with survival in N1–3 subgroup. In TNM II-III subgroup, it was revealed that BMI (P = 0.000), weight loss (P = 0.000) and diabetes (P = 0.000) showed association with longer OS, while weight loss (P = 0.000) was related to survival time in subgroup TNM I stage. To our knowledge, this is the first time that a study has grouped the clinicopathological characteristics to further investigate the effect of MS on ESCC survival.

All in all, our study confirmed that MS patients had a better prognosis in male esophageal cancer patients, not in female. In addition, TNM staging, weight changes and diabetes were also independent predictors for the prognosis of ESCC. The biological mechanisms that could explain the inverse association remain unclear. In the early stage of esophageal cancer, the good control of the blood glucose, lipids, weight and other factors of metabolic sydrome will greatly affect the prognosis.

## Methods

### Patients

A retrospective study was carried out, which included 519 patients with esophageal squamous cell carcinoma who had received esophagetomy and lymphnode dissections in the Department of Thoracic Surgery, Qilu Hospital of Shandong University between January 2007 and December 2011. The study was approved by the Ethical Committee of Qilu Hospital of Shandong University. Informed consent was obtained from all the patients. All data had been anonymized and deidentified. Moreover, the patient data collection methods were carried out in accordance with the Declaration of Helsinki. The patient’s exclusion criteria were as follows: non-primary esophageal cancer, non-first-time diagnosed esophageal cancer, non-squamous cell carcinoma, combined with other malignancies, incomplete clinical pathological information, received neoadjuvant chemoradiotherapy, palliative or R1/R2 resections, lost to follow-up.

### Metabolic syndrome evaluation

There is no consensus on the diagnostic criteria of metabolic syndrome in various groups. In this study, we conducted the diagnosis of MS by the CDS standard proposed by the Chinese medical association diabetes branch.

MS is defined as including three or four of the following criteria: overweight and/or obesity, BMI (Body Mass Index) is greater than 25.0 kg/m^2^; high blood glucose, FPG is greater than 6.1 mmol/L(110 mg/dl) and (or) 2hPG is greater than 7.8 mmol/L (140 mg/dl), and/or has been diagnosed with diabetes; hypertension, SBP/DBP >140/90 mmHg, and/or has been diagnosed with hypertension; dyslipidemia, blood TG is more than 1.7 mmol/L(110 mg/dl), and (or) fasting blood HDL-C <0.9 mmol/L(35 mg/dl) in males, <1.0 mmol/L (39 mg/dl) in females. Little weight loss refers to weight loss within 5%, middle weight loss is 5% to 10%, much weight loss is greater than 10%.

### Follow up

All patients were followed up, including postoperative recovery, general health, necessary psychological support and rehabilitation guidance through telephone, SMS, email, outpatient visit, etc. The information were obtained every 3 months in the first two years, followed by every 6 months. Recurrent, metastatic, lost and dead patients were noted during the follow-up. The time was cut-off until December 2016.

### Statistical analysis

SPSS Version 19.0 software (SPSS Inc., Chicago, IL, USA) was used for statistical analysis. The relationship between MS and various clinical pathological characteristics was tested by the Pearson’s chi square test. OS was the endpoint of the clinical trial, which was defined as the time from the date of the operation to the end of the patient’s death or follow-up. PFS was also one of the endpoints of this study, which was defined as the time from the date of surgery to the progression of the tumor or the end of the patient’s death or follow-up. Kaplan-meier was used to draw the survival curve and the log-rank method was used to test its significance. COX regression included univariate and multivariate analyses. Univariate analysis detected the element related to esophageal cancer survival time, while the independent prognostic factors affecting OS and PFS were determined by multivariate analysis. P < 0.05 was considered statistically significant.

## Electronic supplementary material

Dataset 1
