# Supplementary material for: The prognostic significance of metabolic syndrome and weight loss in esophageal squamous cell carcinoma
Source: Sci Rep. 2018 Jul 4;8:10101. doi: 10.1038/s41598-018-28268-2 (PMC6031687; doi:10.1038/s41598-018-28268-2)
Supplement: Supplementary file 1 — Dataset 1 [file 41598_2018_28268_MOESM1_ESM.docx]

**1. Title :**

The prognostic significance of metabolic syndrome and weight loss in esophageal squamous cell carcinoma

**2. Authors:**

Bowen Liu^1^, Bo Cheng^2^, Cong Wang^3^, Pengxiang Chen^4^, and Yufeng Cheng^5,*^

**Supplementary table S1**

**A correlation analysis between patients with esophageal cancer who are accompanied with obesity, diabetes, dyslipidemia, and hypertension and weight loss**

| Characteristics | Weight loss | | | |
| --- | --- | --- | --- | --- |
|  | No | A little | Middle | Much |
|  | 96 | 89 | 239 | 95 |
| BMI |  |  |  |  |
| ＜18.5 | 8 | 6 | 34 | 38 |
| 18.5~25 | 59 | 64 | 173 | 51 |
| ＞25 | 29 | 19 | 32 | 6 |
| P value | 0.000 |  |  |  |
| Diabetes |  |  |  |  |
| no | 72 | 75 | 195 | 88 |
| yes | 24 | 14 | 44 | 7 |
| P value | 0.012 |  |  |  |
| Dyslipidemia |  |  |  |  |
| no | 62 | 80 | 204 | 88 |
| yes | 34 | 9 | 35 | 7 |
| P value | 0.000 |  |  |  |
| Hypertention |  |  |  |  |
| no | 64 | 70 | 199 | 77 |
| yes | 32 | 19 | 40 | 18 |
| P value | 0.009 |  |  |  |
